# Non-destructive investigation of sandstone blocks used in the Wat Phu temple in Laos and the Banteay Chhmar temple in Cambodia

**DOI:** 10.1016/j.heliyon.2023.e16357

**Published:** 2023-05-17

**Authors:** Etsuo Uchida, Yusu Lu, Lui Du

**Affiliations:** Department of Resources and Environmental Engineering, Faculty of Science and Engineering, Waseda University, Shinjuku, Tokyo 169-8555, Japan

**Keywords:** Wat Phu temple, Banteay Chhmar temple, Sandstone, Magnetic susceptibility, Chemical composition, Non-destructive measurement

## Abstract

Sandstone blocks quarried from the late Jurassic to the early Cretaceous Red Terrane Formation were used to construct the Wat Phu temple in Laos and the Banteay Chhmar temple in Cambodia. The sandstone blocks of the Banteay Chhmar temple are gray to yellowish brown in color and their magnetic susceptibilities and Sr contents are relatively high, similar to the sandstone blocks used in the Angkor monument. In contrast, the Wat Phu temple consists of reddish sandstone blocks with significantly lower magnetic susceptibilities and Sr contents than those used in the Banteay Chhmar temple and Angkor monument. The sandstone blocks of the Banteay Chhmar temple were likely supplied from quarries in Ta Phraya, Thailand, and those of the Wat Phu temple are likely to have been supplied from the area near these temples. The Red Terrane Formation is widely distributed throughout Mainland Indochina, and most of these sandstones show low magnetic susceptibilities and low Sr contents, similar to those of the Wat Phu temple. Sandstone with high magnetic susceptibilities and high Sr contents is found in the sandstone quarries in Ta Phraya and the southeastern foothill of Mt. Kulen, which is the supply source of the sandstone blocks used in the Angkor monument, early buildings of the Bakan monument, and Banteay Chhmar temple. The sandstone with high magnetic susceptibility and high Sr content is distributed in limited areas and implies a weak degree of weathering during the sandstone formation process or a difference in the source rocks.

## 1 Introduction

### 1.1 Purpose of the study

**Fig. 1:**
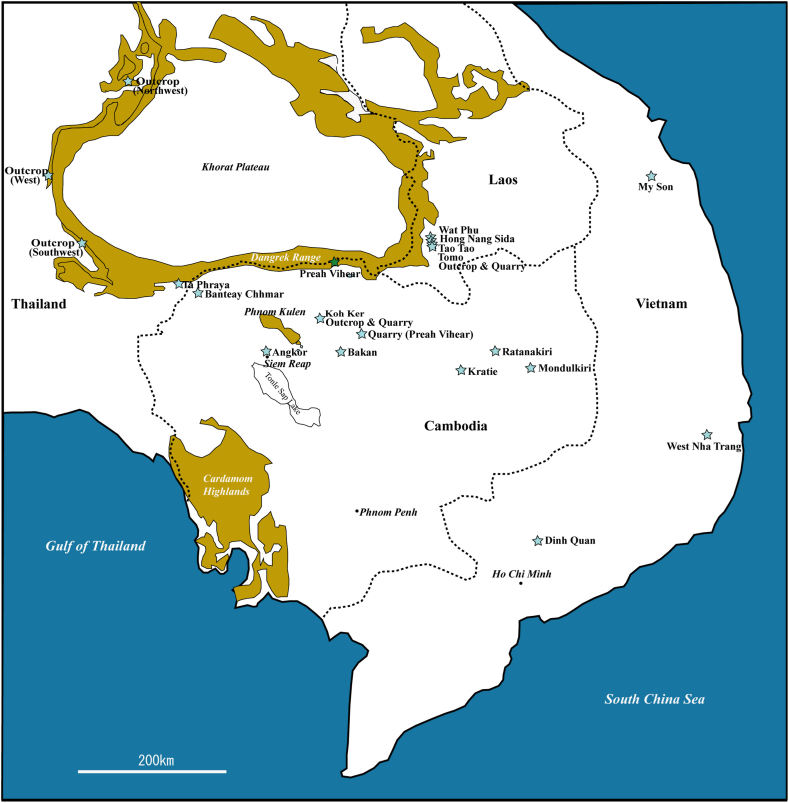
Map of the study area.

Many buildings of the Khmer monuments in Cambodia were constructed using feldspathic arenite (sandstone) quarried from the late Jurassic to early Cretaceous Red Terrane Formation (called the Phu Kradung Formation in Thailand) [1]. The Khmer monuments where such feldspathic arenite is used include the Angkor monument [2,3], the Bakan monument [4], which is also known as the Preah Khan of Kompong Svay, and the Koh Ker monument [5]. In this study, we investigated the sandstone blocks of the Wat Phu temple and its small surrounding temples (Hong Nang Sida, Tao Tao, and Tomo temples) in Laos and the Banteay Chhmar temple in Cambodia, where similar feldspathic arenite blocks are used (Fig. 1).

We also examined the feldspathic arenite in several outcrops of the Red Terrane Formation, which is widely distributed in Mainland Indochina [6,7], and compared it with the sandstone blocks used in the Khmer monuments.

In this study, we report the results of non-destructive magnetic susceptibility measurements and chemical analyses using a portable X-ray fluorescence (pXRF) analyzer on the sandstone blocks of the Wat Phu temple, its surrounding small temples, and the Banteay Chhmar temple. We compare the sandstones (feldspathic arenite) used in these temples with those of other previously investigated monuments (Angkor, Koh Ker, and Bakan monuments) [2,4,5,8,9]. The sandstone (feldspathic arenite) of the Red Terrane Formation was also investigated and its magnetic susceptibility and chemical composition were compared with the sandstone used in the monuments in order to clarify the supply source of the sandstone blocks.

The portable magnetic susceptibility meter is a non-destructive and in-situ investigation method that is extremely useful for clarifying the construction sequence and age of the Angkor monuments built of feldspathic arenite with no differences in chemical composition [8,9]. This method is also useful for identifying the source of supply [10,11]. On the other hand, differences in chemical composition (As, Sr, V, and Y) were found in laterite used in the Angkor monuments and also in the bridges along the East Royal Road. A portable X-ray fluorescence analyzer played an important role in estimating the construction age and the supply range of laterite blocks based on the chemical composition [12,13].

### 1.2 Overview of investigated temples

**Fig. 2:**
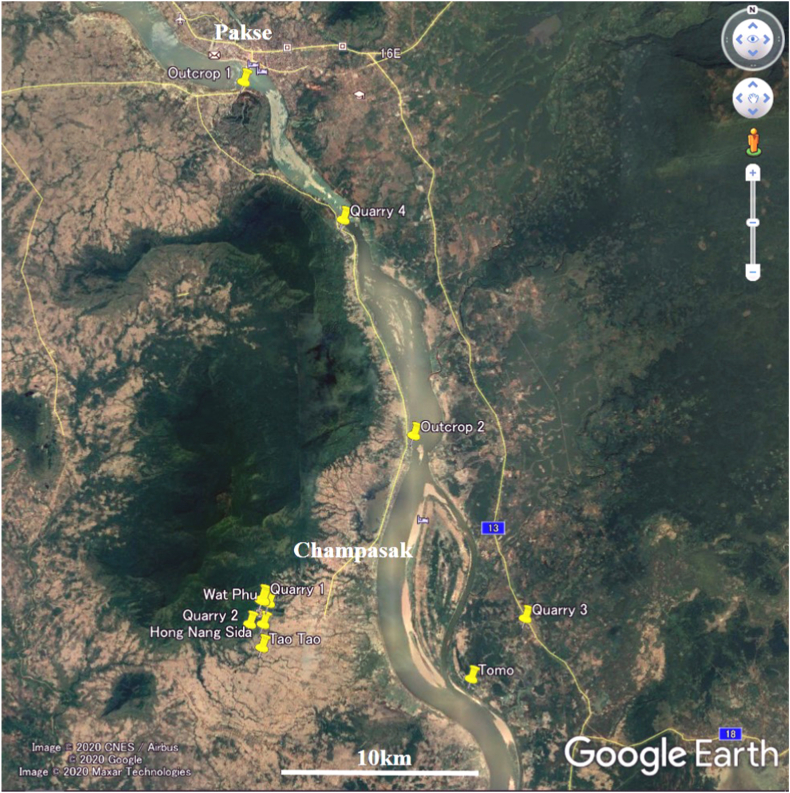
Locations on a Google Earth® image of the investigated Khmer temples, quarries, and outcrops in Pakse and Champasak, Laos.

**Fig. 3:**
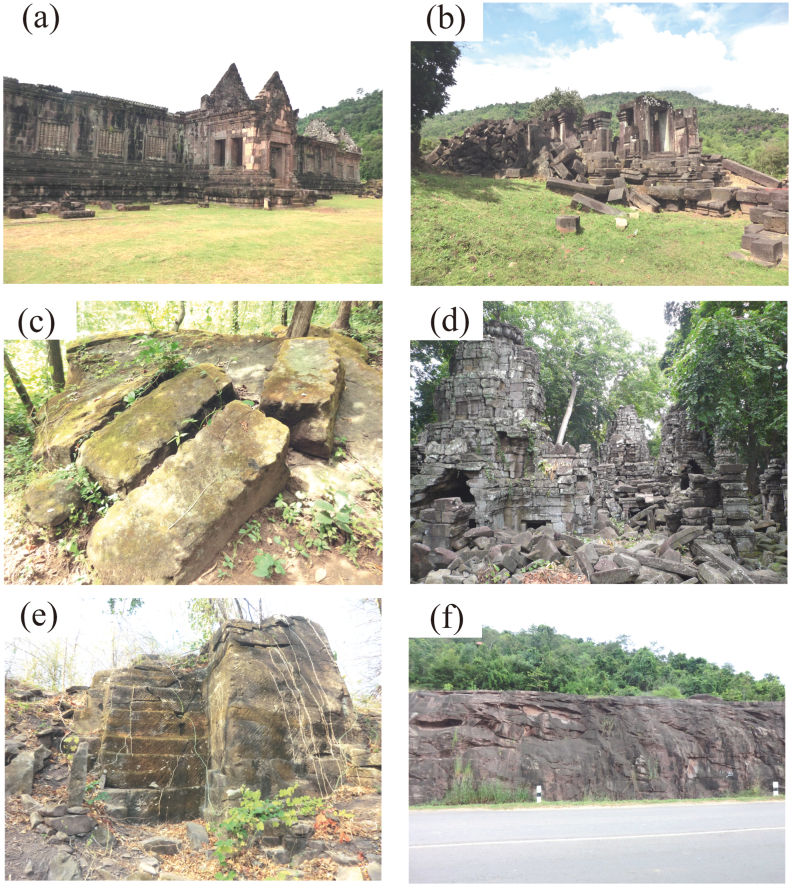
The investigated temples, sandstone quarries, and outcrops: (a) Southern palace of the Wat Phu temple in Laos; (b) Hong Nang Sida temple in Laos; (c) ancient sandstone quarry (Quarry 2) behind the Hong Nang Sida temple in Laos; (d) central area of the Banteay Chhmar temple in Cambodia; (e) ancient sandstone quarry in Ta Phraya in Thailand; and (f) sandstone outcrops (Outcrop 1) on the foothill of Mt. Phu Salao in Laos.

The Wat Phu temple is located in the Champasak region in southern Laos (Fig. 2), 260 km northeast of the Angkor monument. The Wat Phu temple was built on the southeastern foothill of Mt. Phu Kao. The sandstone and laterite buildings of the Wat Phu temple are believed to have been built primarily between the 11th and 13th centuries (Fig. 3a). Part of the top sanctuary is made of brick and its construction is thought to date back to the 10th century or earlier [14]. We also investigated small temples in the vicinity including the Hong Nang Sida (Fig. 3b), Tao Tao, and Tomo temples. These temples were mainly constructed using gray or reddish feldspathic arenite. We therefore also investigated the sandstones in quarries along the southeastern slopes of Mt. Phu Kao (Fig. 3c) to the west of the Wat Phu and Hong Nang Sida temples and on the riverbed near the Tomo temple.

The Banteay Chhmar temple is located 110 km northwest of the Angkor monument and was built in the late Bayon period (late 12th to early 13th centuries) [15]. The Banteay Chhmar temple consists of a central temple (Fig. 3d) surrounded by an outer gallery with dimensions of 220 m in the east-west direction and 180 m in the north-south direction, satellite temples located to the north, south, east, and west, and a fire shrine. Gray to yellowish brown sandstone and laterite were used to build this temple. The sandstone quarry is located in Ta Phraya situated on the southern foothill of the Dangrek Range in Thailand, 25 km west-northwest of the temple, with several stepped quarrying traces at a height of approximately 5–6 m (Fig. 3e). [16]. An approximately 12-km-long canal from the Banteay Chhmar temple toward the sandstone quarry can be seen on Google Earth®, and it is speculated that this canal was used to transport the sandstone blocks.

## 2 Methods

The following non-destructive investigations were carried out on the sandstone (feldspathic arenite) used in the Wat Phu temple, its surrounding temples, and Banteay Chhmar temple, and in outcrops of the Red Terrane Formation.

### 2.1 Chemical composition measurements using a portable XRF

Chemical composition analysis for stone cultural properties by a non-destructive method using a pXRF analyzer is a very useful method for identifying the types of stone materials and for understanding differences in chemical composition [5,13,[17], [18], [19], [20]].

**Fig. 4:**
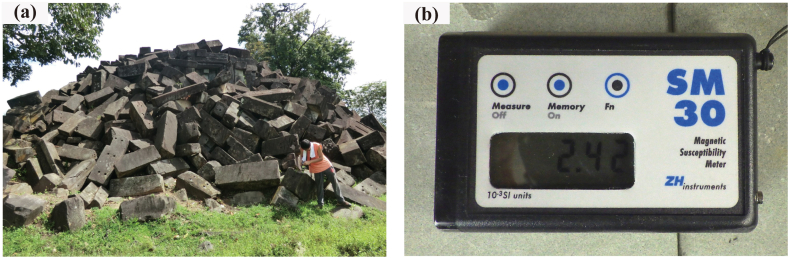
Non-destructive instruments used in this investigation. (a) A portable XRF analyzer at the Hong Nang Sida temple in Laos, and (b) a magnetic susceptibility meter.

Non-destructive chemical analyses of the sandstone blocks used in the Banteay Chhmar, Wat Phu, Hong Nang Sida, Tao Tao, and Tomo temples were conducted using a pXRF analyzer (Delta Premium; Innov-X Systems Inc., Waltham, MA, USA) in a soil mode (Fig. 4). XRF analysis can determine the presence and concentration of a range of metallic and non-metallic elements by measuring the secondary X-rays emitted from a sample when it is excited by X-rays. A calibration of the pXRF analyzer was performed prior to analysis using Japanese standard rock samples (JA-1, JA-2, JB-1b, JB-2, JB-3, JG-1a, JG-2, JGb-1, JR-1, and JR-2) [21]. The accuracy of measurement by the portable XRF analyses is shown in Table 1, Table 2. The total measurement time was set to 60 s. Measurements were conducted on the surfaces of 10 sandstone blocks that were not covered with soil, lichen, or algae from each building, and an average value was calculated. In the sandstone outcrops, the analysis was conducted on flat rock surfaces broken by a hammer. In the sandstone quarries, the analysis was conducted on the flat broken surfaces of fallen sandstone fragments.

**Table 1:** Element concentrations by the portable XRF and magnetic susceptibilities of feldspathic arenite in Laos.

	K (ppm)	Ca (ppm)	Ti (ppm)	Cr (ppm)	Mn (ppm)	Fe (ppm)	Cu (ppm)	Zn (ppm)	Rb (ppm)	Sr (ppm)	Y (ppm)	Zr (ppm)	Pb (ppm)	M.S.^a^
Acccuracy	±286	±60	±40	±8.3	±11	±104	±3.5	±3.1	±1.4	±2.4	±1.0	±2.2	±2.1	±0.001
**Wat Phu: buildings**														
Wat Phu: 1st Terrace	3750	8096	2047	20.3	610	20136	18	57.9	29.0	87.1	14.8	103.7	32.2	0.29
Wat Phu: Causeway	3615	7200	2152	31.0	619	15871	16	28.1	31.1	89.6	14.0	123.5	15.2	0.27
Wat Phu: Southern palace	1763	7421	1598	17.8	506	12049	26	26.6	28.4	101.7	20.3	101.6	9.4	0.26
Wat Phu: Northern palace	3327	7388	2565	28.3	684	17609	25	43.0	32.8	87.5	15.1	168.1	18.1	0.29
Wat Phu :Nandin	1674	6857	1432	13.8	1310	11461	19	25.5	23.3	81.8	14.8	88.7	8.0	0.30
Wat Phu: 2nd Terrace	2350	7149	1514	15.7	522	12080	17	24.7	23.3	79.8	11.3	82.8	10.6	0.24
Wat Phu: 4th Terrace	3832	8577	2340	27.5	797	14219	20	34.7	24.9	96.1	15.8	109.3	14.4	0.28
Wat Phu: Sanctuary	2627	9813	1624	14.9	303	12549	20	26.6	25.5	98.5	14.7	118.7	25.3	0.28
**Wat Phu: Balusters**														
Northern palace (Northern part)	13238	10630	2447	38.5	857	21117	18	43.1	54.9	161.9	18.0	163.2	14.5	0.36
Northern palace (Southern part)	15476	10422	2073	35.4	442	20528	23	39.5	59.1	189.1	18.8	196.0	12.6	0.42
Southern palace (Northern part)	15710	9293	1593	28.4	372	17959	18	33.0	56.7	187.7	16.9	155.2	10.7	0.28
Southern palace (Eastern part)	12444	9132	2338	42.6	802	21635	22	45.4	52.0	166.8	19.6	202.2	14.6	N/A
Southern palace (Western part)	14318	9747	1612	25.0	470	19727	13	38.7	53.7	186.7	17.5	127.7	10.3	N/A
Nandin hall	3266	6864	1549	12.3	467	13591	19	28.8	27.7	83.4	15.1	112.6	8.8	0.20
Sanctuary	4911	9096	2230	19.8	603	17708	42	70.0	29.0	92.1	14.3	190.0	15.2	0.28
**Tao Tao**														
Tao Tao: Sanctuary	9678	10185	1608	26.3	312	13844	14	33.0	43.5	145.7	16.2	113.9	20.4	0.24
Tao Tao: Eastern gopura	3807	10597	1425	16.7	243	12918	14	26.3	25.6	88.4	14.5	87.3	11.6	0.20
**Hong Nang Sida**														
Hong Nang Sida: Mandapa	8565	9168	2245	28.2	327	19969	16	59.3	43.6	120.1	18.1	197.4	19.6	0.31
Hong Nang Sida: Sanctuary	11165	9026	2621	36.3	446	21432	13	61.4	60.2	137.1	16.7	203.6	70.3	0.33
**Tomo**														
Tomo: Southern gopura	5762	7801	2004	17.7	331	12779	31	28.8	29.6	72.1	13.7	103.6	6.4	0.14
**Quarries and Outcrops around Wat Phu**														
Quarry 1: Ancient Quarry behind Sanctuary of Wat Phu (N 14°50' 56.09″, E 105° 48'50.50")	3102	7699	1424	13.0	360	11985	17	28.6	21.7	90.1	9.8	81.2	14.1	0.31
Quarry 2: Ancient Quarry behind Hong Nang Sida (N 14° 50' 18.5″, E 105° 48' 28.9″)	4250	10476	2361	100.7	461	44537	17	49.1	26.2	79.8	11.7	123.7	16.9	0.29
Quarry 3: Ancient Quarry near Tomo (N 14°50' 19.18″, E 105°56' 40.16)	5140	10007	2891	33.8	1604	57286	15	109.0	43.1	116.7	16.8	178.5	9.8	0.21
Quarry 4: Modern Quarry along the road between Champasak and Pakse (N 15° 01' 51.9″, E 105° 51' 28.6″)	11329	8533	1509	17.6	318	15181	14	32.5	42.7	151.1	13.6	112.3	9.2	0.24
Outcrop 1: Outcrop on the foot of Phu Salao (N 15° 05' 51.6″, E 105° 48' 35.0″)	13057	10413	2051	27.1	493	21329	23	69.6	52.5	196.8	16.5	134.7	15.2	0.20
Outcrop 2: Outcrop on the west coast of the Mekong River (N 14°55' 37.85″, E 105°53' 27.28″)	2404	7738	2045	34.4	2471	18093	23	34.6	19.7	69.8	16.7	109.8	8.2	0.16

a Magnetic susceptibility (M.S.) in 10^–3^ SI unit.

**Table 2:** Element concentrations by the portable XRF and magnetic susceptibilities of feldspathic arenite in Thailand, Cambodia, and Vietnam.

	K (ppm)	Ca (ppm)	Ti (ppm)	Cr (ppm)	Mn (ppm)	Fe (ppm)	Cu (ppm)	Zn (ppm)	Rb (ppm)	Sr (ppm)	Y (ppm)	Zr (ppm)	Pb (ppm)	M.S.^a^
Accuracy	±286	±60	±40	±8.3	±11	±104	±3.5	±3.1	±1.4	±2.4	±1.0	±2.2	±2.1	±0.001
**Monument in Cambodia**														
Banteay Chhmar	15410	9857	2859	54	518	22074	14	54	59	189	17	207	46	1.68
**Quarry in Thailand**														
Ta Phraya (N 14° 8'12.08″, E 102°52'31.66″)	17322	16760	3205	42	571	23691	16	48	61	173	20	238	16	1.70
**Quarry and Outcrop in Cambodia**														
Modern quarry in Preah Vihear Province , (N 13° 38' 28.5″, E 105° 01' 03.6″)	21127	12307	2655	50	440	22484	11	38	65.5	218	18.8	157	14	0.51
Ourcrop 1 in Kratie Province (N 13° 17' 33.4″, E 106° 06' 18.6″)	25561	9984	2325	37	456	21327	12	42	74.3	205	16.0	132	15	0.19
Ourcrop 2 in Kratie Province (N 13° 15' 59.2″, E 106° 06' 46.4″)	24611	9456	2088	52	454	19509	13	42	70.8	201	14.0	118	16	0.28
Ourcrop 3 in Kratie Province (N 13° 20' 32.8" , E 106° 05' 34.1″)	18875	13401	2283	32	466	21920	12	40	58.7	197	16.1	166	14	0.25
Outcrop 1 in Mondulkiri Province (N 13° 23' 20.7" , E 106° 23' 50.1″)	16569	9321	2896	51	476	19203	15	48	55.5	208	23.5	217	17	0.13
Outcrop 2 in Mondulkiri Province (N 13° 02' 06.0″, E 107° 04' 16.9″)	23174	7059	2494	30	361	19080	13	34	55.9	99	19.2	148	10	0.11
Outcrop 3 in Mondulkiri Province (N 13° 09' 02.9″, E 106° 56' 53.9″)	14670	11330	3331	55	422	22108	14	42	46.5	178	25.1	225	16	0.34
Outcrop in Ratanakiri Province (N 13° 11' 29.7″, E 106° 57' 09.6″)	28661	7713	3246	75	491	21554	12	52	71.7	133	18.3	168	13	0.41
**Monument and Outcrop in Vietnam**														
My Son monuments	11523	8709	1380	33	436	16595	97	41	54.8	229	18.5	185	11	0.267
Outcrop in Nha Trang West (N 12° 16' 15.8″, E 108° 50' 22.4″)	17954	7728	3874	46	332	20977	28	59	61.4	135	34	618	12	0.165
Outcrop in Dinh Quan (N 11° 04' 54.5″, E 107° 19' 54.5″)	25235	7376	3889	51	346	27340	20	56	88.4	113	25	239	11	0.175
**Outcrop in Thailand**														
Outcrop in the southwest of Khorat Plateau (N 14° 25' 32.62″ N, 101° 51' 58.23″ E)	10700	23574	2553	30	872	19721	22	121	50.2	187	22	169	10	0.160
Outcrop in the west of Khorat Plateau (N 14° 48' 05.62″, E 101° 32' 15.07″)	7706	20640	3166	23	461	24164	26	74	42.1	117	21	230	7	0.629
Outcrop in the northwest of Khorat Plateau (N 16° 48' 03.35″, E 102° 36' 52.32″)	10333	15511	2386	29	587	14487	23	30	49.4	75	23	316	8	0.121

a Magnetic susceptibility (M.S.) in 10^–3^ SI unit.

### 2.2 Magnetic susceptibility measurement

Stones with the same chemical composition can often have different magnetic susceptibilities. Magnetic susceptibility measurements can be performed quickly and non-destructively, and can be a very effective technique for clarifying differences in stone materials used in cultural properties [2,8,9,17,18].

Non-destructive magnetic susceptibility measurements were conducted on the sandstone blocks used in the Banteay Chhmar, Wat Phu, Hong Nang Sida, Tao Tao, and Tomo temples using a portable magnetic susceptibility meter (SM30; ZH Instruments, Brno, Czech Republic) (Fig. 4). The magnetic susceptibility meter contains an oscillator with a pickup coil. The change in frequency is proportional to the amount of magnetic susceptibility of the rock. In order to find out about the change, it is necessary to measure the oscillator frequency twice. The first measurement is performed on the surface of the rock. The second measurement is carried out when the meter is away from the rock. After the second step is finished, both values are subtracted and displayed (mode 1) (the user's manual of the magnetic susceptibility meter SM30). We measured 5 × 5 cm sandstone block surfaces. The measurement time was approximately 2 s and an accuracy of 1 × 10^−6^ SI units was obtained. Magnetic susceptibility measurements were made on 50 sandstone blocks at each building and an average value was calculated. Measurements were carried out on the flat surfaces of sandstone blocks not covered with soil, lichen, or algae. Magnetite is considered to be the main reason for the magnetic susceptibility of the sandstone.

## 3 Results

### 3.1 Wat Phu temple and its surrounding temples

#### 3.1.1 Magnetic susceptibility

**Fig. 5:**
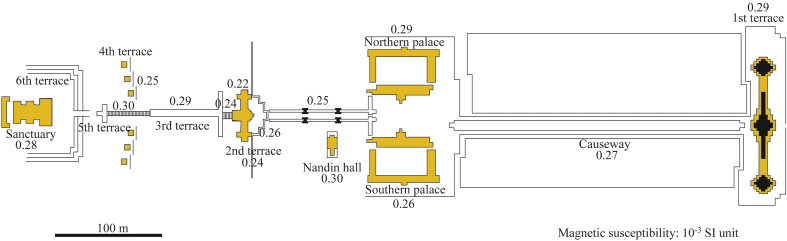
Plan of the Wat Phu temple, showing the distribution of magnetic susceptibilities (10^−3^ SI units) of the sandstone blocks.

The magnetic susceptibility of the sandstone blocks used in the Wat Phu (Fig. 5), Hong Nang Sida, Tao Tao, and Tomo temples ranges from 0.14 to 0.42 × 10^−3^ SI units and mainly between 0.20 and 0.32 × 10^−3^ SI units (Table 1) (Supplementary Material 1). However, the magnetic susceptibilities of the balusters in the northern and southern palaces of the Wat Phu temple are slightly higher (0.36× 10^−3^ and 0.42 × 10^−3^ SI units, respectively) than the sandstone blocks used for buildings in the investigated temples (Table 1). This suggests that the sandstone blocks used for the balusters in the palaces of the Wat Phu temple were supplied from different quarries from those of the buildings in the Wat Phu temple.

#### 3.1.2 Chemical compositions

The following elements were detected in all of the sandstone blocks: K, Ca, Ti, Cr, Mn, Fe, Cu, Zn, Rb, Sr, Y, Zr, and Pb (Table 1) (Supplementary Material 2). Systematic differences were identified in the Sr and Rb contents. The sandstone blocks in the following locations are relatively rich in Sr and Rb: balusters in the northern (176 and 57 ppm, respectively) and southern (188 and 57 ppm) palaces of the Wat Phu temple, the sanctuary of the Tao Tao temple (146 and 44 ppm), and the Hong Nang Sida temple (129 and 59 ppm). In the other locations, the Sr and Rb contents are low and the average values are 110 and 35 ppm or less, respectively. The K content also tends to be low (1700–3800 ppm).

### 3.2 Banteay Chhmar temple

#### 3.2.1 Magnetic susceptibility

**Fig. 6:**
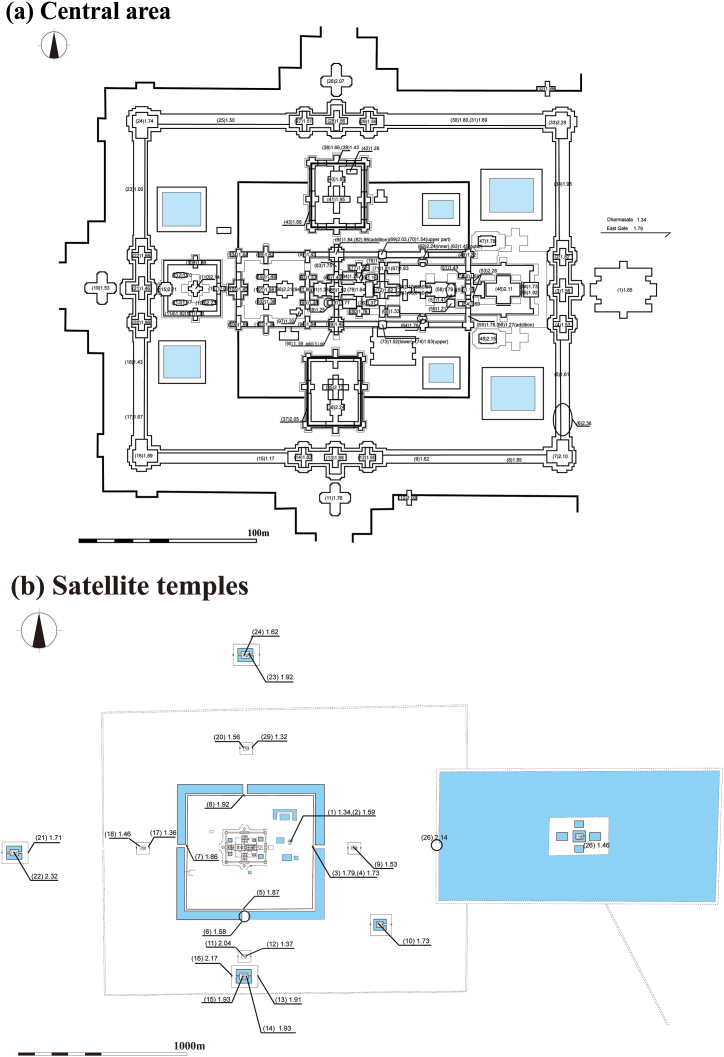
Plan of the Banteay Chhmar temple, showing the distribution of magnetic susceptibilities (10^−3^ SI units) of the sandstone blocks. (a) Central area, and (b) satellite temples.

Magnetic susceptibility measurements were performed at a total of 141 locations. Fig. 6 shows the measurement locations and average magnetic susceptibility at each location. The full measurement results are shown in Supplementary Material 3. The measurements were made on 50 sandstone blocks at each location. The minimum and maximum average values were 0.94 × 10^−3^ and 2.36 × 10^−3^ SI units, respectively, and the overall average was 1.68 × 10^−3^ SI units (Table 2). At the Bayon, Ta Prohm, Preah Khan, and Banteay Kdei temples in the Angkor monument, which were built during the same Bayon period, the magnetic susceptibility of the sandstone blocks changes systematically over time and it was therefore possible to estimate the order of construction based on the magnetic susceptibility [8]. However, in the case of the Banteay Chhmar temple, no systematic change in magnetic susceptibility was observed and thus the order of construction could not be estimated.

Magnetic susceptibility was also measured at quarries in Ta Phraya, Thailand (Fig. 3f), which is considered to be the supply source for the Banteay Chhmar temple sandstone. Large quarrying traces were found in four locations with average magnetic susceptibilities of 1.42, 1.20, 2.05, and 1.76 × 10^−3^ SI units, respectively, and 1.70 × 10^−3^ SI units in total (Supplementary Material 4). This value is nearly the same as the average magnetic susceptibility for the sandstone blocks of the Banteay Chhmar temple (1.68× 10^−3^ SI units).

**Fig. 7:**
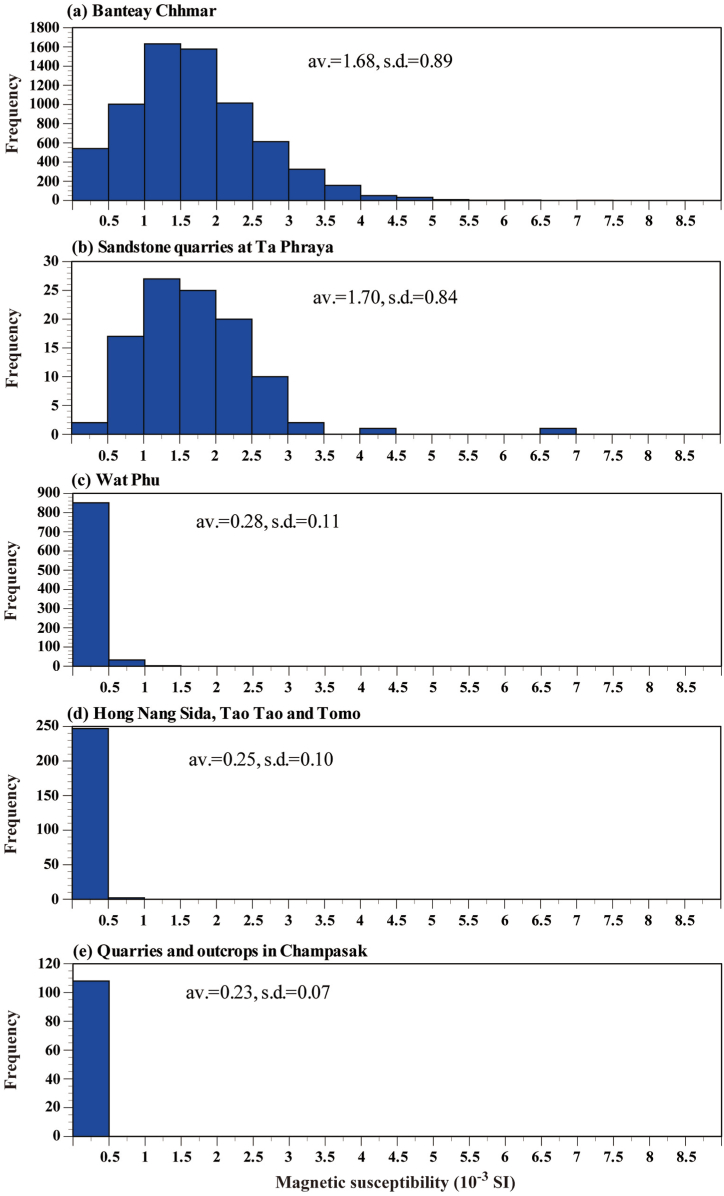
Frequency diagrams of the magnetic susceptibilities of the sandstones. (a) Banteay Chhmar temple, (b) sandstone quarries in Ta Phraya, (c) Wat Phu temple, (d) Hong Nang Sida, Tao Tao and Tomo temples, and (e) quarries and outcrops in Champasak.

Fig. 7 shows a frequency diagram of the magnetic susceptibility. Although the number of measurements at the Ta Phraya quarries is small, the sandstone at the Banteay Chhmar temple and Ta Phraya quarries show similar shaped frequency diagrams. This indicates that the Ta Phraya quarries are likely to be the host quarries of the Banteay Chhmar temple sandstone.

#### 3.2.2 Chemical composition

There are no notable differences between the sandstone of the Banteay Chhmar temple and that of the Angkor, Bakan, and Koh Ker monuments, with Sr and Rb contents in the range of approximately 173–220 and 50–82 ppm, respectively. (Table 2) (Supplementary Material 5).

The surface of the sandstone at the quarries in Ta Phraya is severely altered. Sandstone fragments that had fallen around the quarries were thus broken with a hammer and cross sections were analyzed using a pXRF analyzer (Table 2) (Supplementary Material 4). The measured Sr and Rb contents were in the range of approximately 154–195 and 49–86 ppm, respectively. The Rb content was similar to that of the Banteay Chhmar temple sandstone, but the Sr content was approximately 25 ppm lower on average. Considering that Sr tends to be leached more rapidly by weathering than Rb, the chemical composition of the Ta Phraya sandstone can be considered to be nearly the same as that of the Banteay Chhmar temple sandstone.

### 3.3 Outcrops and quarries of sandstone in the Red Terrane Formation

#### 3.3.1 Sandstone outcrops and quarries of the Red Terrane Formation in Laos

Magnetic susceptibility measurements and pXRF analyses were conducted in the following sites (Fig. 2): Quarry 1: ancient quarry behind the sanctuary of the Wat Phu temple, Quarry 2: ancient quarry behind the Hong Nang Sida temple (Fig. 3c), Quarry 3: ancient quarry along the river near the Tomo temple, Quarry 4: modern quarry along the road between Champasak and Pakse, Outcrop 1: outcrop on the foothill of Mt. Phu Salao in Pakse, and Outcrop 2: outcrop on the west coast of the Mekong River in Champasak.

The magnetic susceptibility of the sandstones in Quarries 1–4 and Outcrops 1 and 2 range from 0.16 to 0.31 × 10^−3^ SI units (Table 1) (Supplementary Material 1). These values are similar to those of the sandstone blocks used in the investigated temples in Champasak.

The Sr and Rb contents of the sandstones are relatively low in Quarry 1 (90 and 22 ppm, respectively), Quarry 2 (80 and 26 ppm), and Outcrop 2 (70 and 20 ppm) (Table 1). In contrast, these values are relatively high in Outcrop 1 (197 and 53 ppm), Quarry 3 (117 and 43 ppm), and Quarry 4 (151 and 43 ppm) (Table 1) (Supplementary Material 2). Aside from Quarry 4 and Outcrop 1, the K contents tend to be low (2400–5200 ppm).

#### 3.3.2 Sandstone outcrops of the Red Terrane Formation in Cambodia

Measurements of sandstone in the Red Terrane Formation were conducted at one modern quarry in Preah Vihear province, three outcrops in Kratie province, three outcrops in Mondulkiri province, and one outcrop in Ratanakiri province in Cambodia (Table 2) (Supplementary Material 5). The modern quarry in Preah Vihear province is located 38 km northeast of the Bakan monument and contains sandstone with mostly low average magnetic susceptibility (0.51× 10^−3^ SI units), although some areas with high magnetic susceptibility were also noted. These average values are low compared with the sandstone blocks of the Angkor monument. However, the average Sr and Rb contents are 218 and 66 ppm, respectively, which are similar to those of the Angkor monument sandstone. The average magnetic susceptibility of the sandstone at the outcrops in Kratie, Mondulkiri and Ratanakiri provinces were as low as 0.24, 0.19, and 0.41 × 10^−3^ SI units, respectively, the average Sr contents were 201, 162, and 133 ppm, and the average Rb contents were 68, 53, and 72 ppm. The magnetic susceptibility in all places was lower than that of the sandstone in the Angkor monument. The outcropped sandstones in Kratie province showed similar Sr and Rb values to the Angkor monument sandstone. The Rb contents in the sandstones of Mondulkiri and Ratanakiri provinces were similar to those of the Angkor monument, whereas the Sr contents were slightly lower.

#### 3.3.3 Sandstone in the My Son monument and sandstone outcrops in Vietnam

Measurements were made on the sandstone blocks used in the B1 building, which is the only sandstone structure in the My Son monument, and those in the openings of other buildings. Measurements were also made on the sandstone outcrops of the Red Terrane Formation approximately 44 km west of Nha Trang City and 75 km northeast of Ho Chi Minh City (Table 2) (Supplementary Material 5). The magnetic susceptibilities of the sandstone at these three sites were relatively low with average values of 0.27, 0.16, and 0.17 × 10^−3^ SI units, respectively, average Sr contents of 202, 135, and 113 ppm, and Rb contents of 56, 61, and 88 ppm. The Rb contents were approximately the same as the Angkor monument sandstone. The Sr contents of the My Son monument sandstone were essentially the same as the Angkor monument sandstone, whereas the values at the outcrops were lower.

#### 3.3.4 Sandstone outcrops of the Red Terrane Formation in Thailand

Measurements were made at three locations (southwest, west, and northwest) on the sandstone of the Red Terrane Formation (Phu Kradung Formation) distributed in the western areas of the Khorat Plateau [22] (Table 2) (Supplementary Material 5). The average magnetic susceptibility showed low values of 0.16, 0.63, and 0.12 × 10^−3^ SI units, respectively, with average Sr contents of 187, 117, and 75 ppm, and average Rb contents of 50, 42, and 49 ppm. The sandstone in the southeastern area showed nearly the same composition as the Angkor monument sandstone, but the Sr contents were low in the other areas.

## 4 Discussion

### 4.1 Magnetic susceptibility

The magnetic susceptibilities of the sandstone blocks used in the Banteay Chhmar temple as well as the Angkor monuments [2,8,9] are higher than those used in the Wat Phu, Hong Nang Sida, Tao Tao and Tomo temples. The magnetic susceptibility of the sandstone blocks in the Banteay Chhmar temple is relatively high and ranges from 0.9 to 2.4 × 10^−3^ SI units (Fig. 6). These values are similar to those of the sandstone blocks in the Angkor monuments (0.7 to 8.8 × 10^−3^ SI units) and the early-stage buildings of the Bakan monument (1.4 to 2.0 × 10^−3^ SI units). The sandstone blocks with relatively high magnetic susceptibilities ranging from 0.7 to 1.3 × 10^−3^ SI units are also used in the Koh Ker monument [5]. The sandstone blocks used in the Angkor monument and early-stage buildings of the Bakan monument are deduced to have been supplied from the southeastern foothill of Mt. Kulen [4], where many traces of ancient sandstone quarries remain [10,11,[23], [24], [25], [26], [27], [28]]. The magnetic susceptibilities of the sandstone blocks in the Wat Phu temple and its surrounding temples (0.14 to 0.42 × 10^−3^ SI units) are remarkably lower than those used in the Angkor monuments, the Banteay Chhmar temple, and the early-stage buildings of the Bakan monument. The Red Terrane Formation is widely distributed in Cambodia, Thailand, Laos, and Vietnam. Except for the southeastern foothill of Mt. Kulen, and the southern foothill of the Dangrek Range where the Ta Phraya sandstone quarry is situated, the sandstone of the Red Terrane Formation shows low magnetic susceptibility, ranging from 0.2 to 0.5 × 10^−3^ SI units.

### 4.2 Chemical composition

The Sr and Rb contents of the sandstone blocks in the Banteay Chhmar temple are 173–220 and 50–82 ppm, respectively, which are similar to those in the Angkor, Koh Ker, and Bakan monuments (Supplementary Material 5). However, the Sr (72–189 ppm) and Rb (23–60 ppm) contents of the sandstones used in the Wat Phu temple and its surrounding temples are lower than the above-mentioned monuments and temples (Table 2).

Judging from these trends, the higher magnetic susceptibility and higher Sr and Rb contents of the sandstone used in the Angkor monument, early-stage buildings of the Bakan monument, supplied from the southeastern foothill of Mt. Kulen, and Banteay Chhmar temple, supplied from Ta Phraya in the southern foothill of the Dangrek Range, seem to be rather exceptional.

### 4.3 Relationship between magnetic susceptibility and chemical composition

**Fig. 8:**
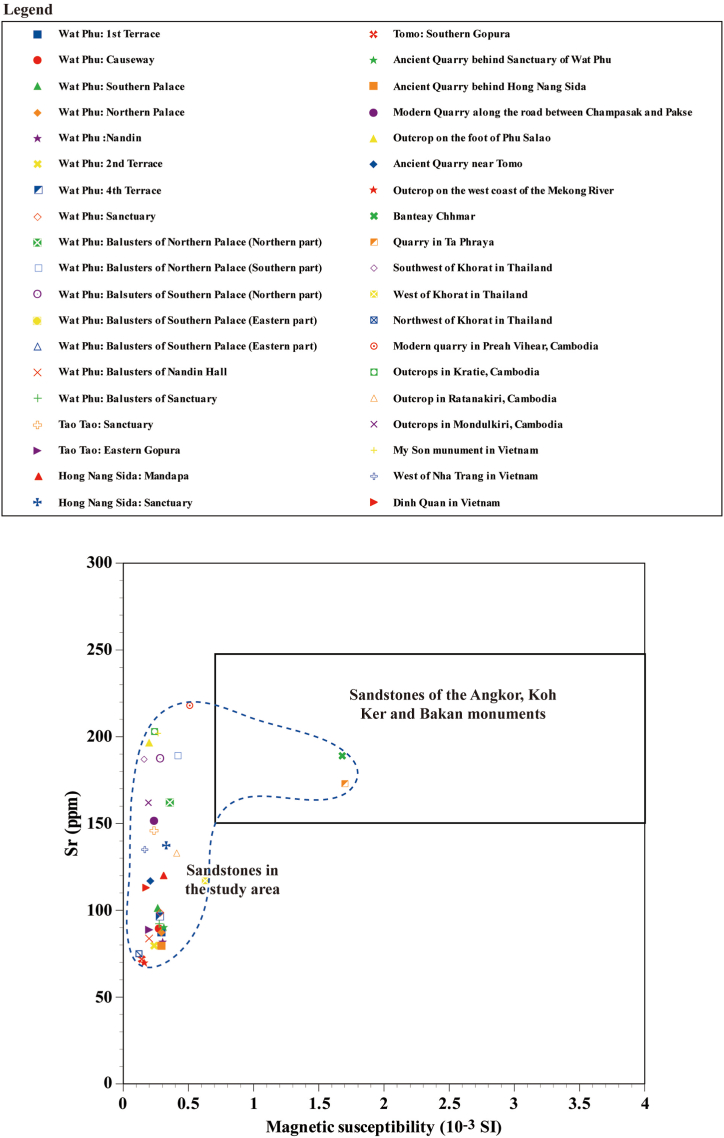
Sr versus magnetic susceptibility diagram for the sandstones in the studied monuments, quarries, and outcrops.

**Fig. 9:**
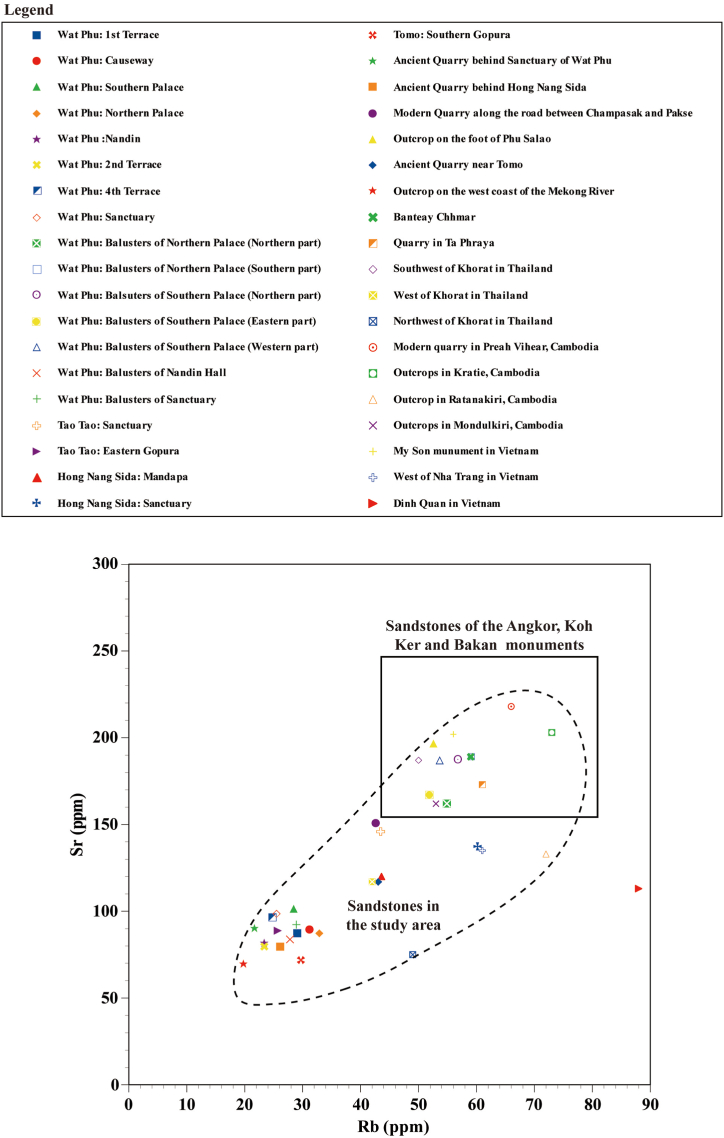
Sr versus Rb diagram for the sandstones in the studied monuments, quarries, and outcrops.

**Fig. 10:**
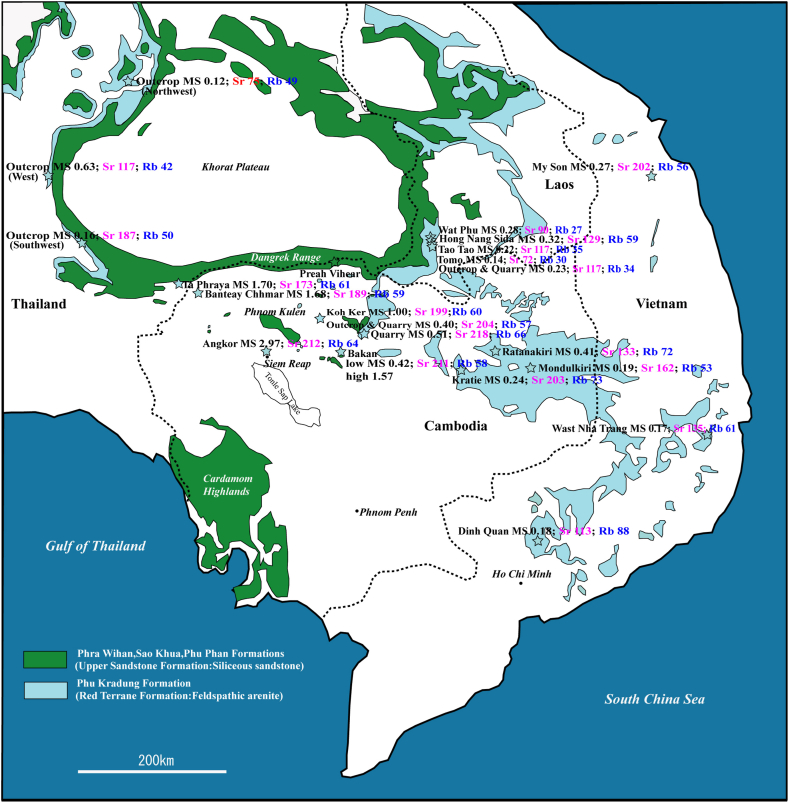
Magnetic susceptibilities (10^−3^ SI units) and Sr and Rb contents (ppm) of sandstone (feldspathic arenite) in the monuments, quarries, and outcrops in Cambodia, Thailand, Laos, and Vietnam.

The sandstone of the Red Terrane Formation of the western (Thailand) and eastern areas (Laos) of the Khorat Plateau, Kratie, Mondulkiri and Ratanakiri provinces in Cambodia, and Nha Trang and Dinh Quan areas in Vietnam shows low magnetic susceptibility and low Sr contents (Fig. 8, Fig. 9, Fig. 10). The magnetic susceptibility and Sr contents of the sandstone of the Red Terrane Formation in these locations show similar trends to the sandstones of the Wat Phu, Hong Nang Sida, Tao Tao and Tomo temples. In contrast, only the sandstone quarries in Ta Phraya and the southeastern foothill of Mt. Kulen show high magnetic susceptibility and high Sr contents. Sandstone with high Sr but with low magnetic susceptibility is observed, for example, in the modern quarry in Preah Vihear province in Cambodia, some outcrops in Kratie province in Cambodia, the My Son monument in Vietnam, the balusters of the palaces of the Wat Phu temple in Laos, and the outcrops in the southwestern part of the Khorat Plateau in Thailand.

As described above, the sandstone of the Red Terrane Formation ranges from high to low magnetic susceptibility and Sr contents, but the Sr content tends to decrease later than the magnetic susceptibility (Fig. 8). That is, the sandstone of the Red Terrane Formation with high Sr contents of 170–250 ppm is observed even though the magnetic susceptibility shows a low value of 0.2 to 0.5 × 10^−3^ SI units. The magnetic susceptibility of the sandstone is considered to be mainly proportional to the amount of magnetite contained therein, whereas Sr is presumed to be mainly contained in plagioclase. Sandstone forms when source rocks are weathered and sand grains are transported by water currents, deposited, and lithified. The above-mentioned measurement results for the sandstone of the Red Terrane Formation seem to indicate that magnetite weathering is more likely to proceed than plagioclase weathering. Conversely, the sandstone with high magnetic susceptibility shows high Sr contents (170–250 ppm). A similar tendency is seen for Rb (Fig. 9) but is slightly less clear. Rb is considered to be mainly contained in K-feldspar, which is considered to be likely more slowly weathered than plagioclase. From this assessment, it can be concluded that the sandstone of the Red Terrane Formation with high magnetic susceptibility and high Sr contents was weakly weathered during its formation process and conversely, the sandstone with low magnetic susceptibility and Sr contents likely formed under the strong weathering process.

### 4.4 Formation condition and sediment source of the sandstone

As mentioned above, stronger weathering during the sandstone formation process is considered to have resulted in the lower magnetic susceptibility and Sr contents of the sandstone of the Red Terrane Formation. Most of the sandstone is therefore considered to have been strongly weathered during its formation. Such sandstone is commonly observed in wide areas of Thailand, Cambodia, Laos, and Vietnam. In contrast, the sandstones at quarries on the southeastern foothill of Mt. Kulen in Cambodia and those in Ta Phraya on the southern foothill of the Dangrek Range in Thailand have exceptionally high magnetic susceptibility and Sr contents compared with other regions. This may indicate that weathering during the sandstone formation process was relatively weak in these areas. In addition, because the Red Terrane Formation is distributed over a very wide area of 1200 km north-south and 800 km east-west in Thailand, Cambodia, Laos, and Vietnam, it is likely that the sediment source also differed from place to place. It is therefore possible that magnetite was abundant in the source rocks of the sandstone as one factor of high magnetic susceptibility in Mt. Kulen and Ta Phraya areas. The results presented here clearly indicate the differences of these particular areas compared with other areas of the study location.

## 5 Conclusions

Non-destructive magnetic susceptibility measurements and chemical analyses using a pXRF analyzer were conducted on the sandstone blocks of the Wat Phu temple, its surrounding small temples, and the Banteay Chhmar temple. The sandstone blocks of the above-mentioned temples were supplied from the Red Terrane Formation. The sandstone of this Formation, distributed throughout the Mainland Indochina, was also investigated, and its magnetic susceptibility and chemical composition were compared with the sandstone blocks used in the temples. We elucidated that most of these sandstones show low magnetic susceptibilities and low Sr contents, similar to those of the Wat Phu temple and its surrounding temples. Most of the sandstone of the Red Terrane Formation is therefore considered to have been strongly weathered during its formation process. On the contrary, sandstone with high magnetic susceptibilities and high Sr contents is found in the sandstone quarries of Ta Phraya and the southeastern foothill of Mt. Kulen, which are the supply source of the sandstone blocks used in Banteay Chhmar temple and the Angkor monument, respectively. The sandstone with high magnetic susceptibility and high Sr content is distributed in limited areas and implies a weak degree of weathering during the sandstone formation process or a difference in the source rocks.

## Author contribution statement

Etsuo Uchida: Conceived and designed the experiments, Performed the experiments, Analyzed and interpreted the data, Wrote the paper.

Yu Sulu & Rui Du: Performed the experiments, Analyzed and interpreted the data.

## Data availability statement

Data included in article/supp. material/referenced in article.

## Declaration of competing interest

The authors declare that they have no known competing financial interests or personal relationships that could have appeared to influence the work reported in this paper.

## References

[1] Meesook A., Ridd M.F., Barber A.J., Crow M.J. (2011). The Geology of Thailand.

[2] Uchida E., Ogawa Y., Nakagawa T. (1998). The stone materials of the Angkor monuments, Cambodia – the magnetic susceptibility and the orientation of the bedding plane of the sandstone. J. Min. Pet. Econ. Geol..

[3] Uchida E., Ogawa Y., Maeda N., Nakagawa T. (1999). Deterioration of stone materials in the Angkor monuments. Cambodia. Eng. Geol..

[4] Uchida E., Shimoda I., Shimoda M. (2013). Consideration of the construction period of the Khmer temples along the east royal road to Preah Khan of Kompong Svay and the provenance of sandstone blocks based on their magnetic susceptibility. Archaeol. Discov..

[5] Uchida E., Tsuda K., Shimoda I. (2014). Construction sequence of the Koh Ker monuments in Cambodia deduced from the chemical composition and magnetic susceptibility of its laterite. Her. Sci..

[6] Tien P.C., An L.D., Bach L.D., Bac D.D., Vongdara B., Phengthavongsa B., Danh T., Dy N.D., Dung H.T., Hai T.Q., Khuc V., Kun S.C., Long P.D., Ly M.N., My N.Q., Ngan P.K., Ngoc N., Ratanavong N., Quoc N.K., Quyen N.V., Aphaymani S.D., Thanh T.D., Tri T.V., Truyen M.T., Xay T.S. (1990).

[7] Mantajit N., Hinthon C. (1990).

[8] Uchida E., Cunin O., Shimoda I., Suda C., Nakagawa T. (2003). The construction process of the Angkor monuments elucidated by the magnetic susceptibility of sandstone. Archaeometry.

[9] Uchida E., Cunin O., Suda C., Ueno A., Nakagawa T. (2007). Consideration on the construction process and the sandstone quarries during Angkor period based on the magnetic susceptibility. J. Archaeol. Sci..

[10] Uchida E., Shimoda I. (2013). Quarries and transportation routes of Angkor monument sandstone blocks. J. Archaeol. Sci..

[11] Uchida E., Watanabe R., Murasugi M., Sakurai Y., Shimoda I. (2020). The sandstone quarries of the Angkor monuments in the southeastern foothills of Kulen Mountain. Archaeol. Discov..

[12] Uchida E., Maeda N., Nakagawa T. (1999). The laterites of the Angkor monuments, Cambodia. The grouping of the monuments on the basis of the laterites. J. Petrol Miner. Econ. Geol..

[13] Uchida E., Sakurai Y., Cheng C., Shimoda I., Saito Y. (2020). Supply ranges of stone blocks used in masonry bridges and their construction period along the East Royal Road in the Khmer Empire, Cambodia. Her. Sci..

[14] Jacques C., Lafond P. (2007).

[15] Sharrock P.D. (2015).

[16] Mollerup A. (2012).

[17] Williams-Thorpe O., Potts J.P., West M. (2008). Portable X-Ray Fluorescence Spectrometry. Capabilities for in Situ Analysis.

[18] Uchida E., Mizoguchi A., Sato H., Shimoda I., Watanabe R. (2017). Determining the construction sequence of the Preah Vihear monument in Cambodia from its sandstone block characteristics. Her. Sci..

[19] Uchida E., Watanabe R., Cheng R., Nakamura Y., Takeyama T. (2021). Non-destructive in-situ classification of sandstones used in the Angkor monuments of Cambodia using a portable X-ray fluorescence analyzer and magnetic susceptibility meter. J. Archaeol. Sci. Rep..

[20] Shimoda I., Uchida E., Tsuda K. (2019). Estimated construction order of the major shrines of Sambor Prei Kuk based on an analysis of bricks. Heritage.

[21] Imai N., Terashima S., Itoh S., Ando A. (1995). 1994 compilation values for GSJ reference samples, “Igneous rock series”. Geochem. J..

[22] Uchida E., Ito K., Shimizu N. (2010). Provenance of the sandstone used in the construction of the Khmer monuments in Thailand. Archaeometry.

[23] Garnier F. (1873).

[24] Delaporte L. (1880).

[25] Delvert J. (1963). Recherches sur l’erosion des grès des monuments d'Angkor. Bull. École Française d’Extrême-Orient.

[26] Boulbet J. (1979).

[27] Carò F., Im S. (2012). Khmer sandstone quarries of Kulen Mountain and Koh Ker: a petrographic and geochemical study. J. Archaeol. Sci..

[28] Evans D. (2016). Airborne laser scanning as a method for exploring long-term socio-ecological dynamics in Cambodia. J. Archaeol. Sci..

